# Clinical application of human umbilical cord-derived mesenchymal stem cells for small articular cartilage defects of the knee in military training-related injuries

**DOI:** 10.12669/pjms.41.8.10803

**Published:** 2025-08

**Authors:** Gen Zhao, Chenchen Xue, Bao Li, Wenming Jin, Xinwei Liu

**Affiliations:** 1 Gen Zhao Department of Orthopedics, General Hospital of the Yangtze River Shipping and Wuhan Brain Hospital, Wuhan 430010, PR China; 2 Chenchen Xue Department of orthopedic surgery, The First Affiliated Hospital of the Naval Medical University, Shanghai 200433, China; 3 Bao Li Department of Orthopaedics, The General Hospital of Northern Theater Command, Shenyang 110016, Liaoning, China; 4 Wenming Jin Department of Orthopaedics, The General Hospital of Northern Theater Command, Shenyang 110016, Liaoning, China; 5 Xinwei Liu Department of Orthopaedics, The General Hospital of Northern Theater Command, Shenyang 110016, Liaoning, China

**Keywords:** Articular cartilage injury of the knee, Human umbilical cord-derived mesenchymal stem cell, Military training-related injury, Microfracture

## Abstract

**Objective:**

To explore the short-term efficacy and safety of umbilical cord-derived mesenchymal stem cells(UC-MSCs) in patients with small articular cartilage defects of the knee.

**Methods:**

This was a retrospective study from August 2020 to December 2020, thirty two patients with knee cartilage injuries caused by military training were admitted to the Department of Orthopedics in The General Hospital of North Theater Command Training Base included in the study. Group-A (n=11) underwent microfracture surgery, Group-B(n=10) underwent arthroscopic exploration and injected umbilical cord mesenchymal stem cells (UC MSCs) into the knee joint cavity, and Group-C (n=11) underwent microfracture surgery and injected UC MSCs into the knee joint cavity. Clinical data were collected from three groups of patients.

**Results:**

The Lysholm scores of the three groups gradually increased, from 51.00 ± 1.6, 51.50 ± 2.2, and 50.91 ± 2.4 points before treatment to 74.64 ± 2.4, 79.40 ± 4.3, and 78.73 ± 4.2 points at the last follow-up (P<0.05), respectively. The AKSS scores also showed similar therapeutic effects. During the follow-up of six, nine, and 12 months after treatment, the scores of Group-B and C were higher than Group-A (P<0.05), while Groups-B and C had similar efficacy(P>0.05).

**Conclusion:**

The application of microfracture combined with UC MSCs injection or UC MSCs injection alone for small cartilage defects in the knee joint can achieve certain therapeutic effects in the short term, but as a new technology, stem cells need further observation for long-term efficacy.

## INTRODUCTION

With the continuous improvement of military training level during peacetime, knee cartilage injury has become very common. If not treated in a timely and effective manner, it is highly likely to develop knee arthritis.^1^ According to statistics, about 80% of knee osteoarthritis (KOA) patients worldwide have limited mobility, of which 25% can lead to disability.^2^ The effectiveness of surgical treatment for knee cartilage injury largely depends on the size and location of the cartilage defect.^3^ Microfractures are a common bone marrow stimulation technique for treating cartilage defects smaller than 2cm.^3^-^5^

It has been reported that within 2~5 years after surgery, the success rate of microfractures in relieving pain is 90%,^6^ Its limitation lies in insufficient wear resistance. In recent years, the progress of stem cell research has provided new ideas for the treatment of cartilage defects. microfracture surgery is more commonly used for cartilage defects ≤ 2 cm², and has shown good therapeutic effects. However, its long-term efficacy is limited by the formation of fibrocartilage which lacks the biomechanical properties of native hyaline cartilage. Moreover, emerging stem cell therapies, particularly umbilical cord-derived mesenchymal stem cells(UC-MSCs), have shown promise in cartilage regeneration but their application in small defects specifically related to military training injuries remains understudied. Military training imposes high mechanical stress on knee joints, demanding treatments that enable rapid return to duty. Current options for small cartilage defects-primarily microfracture-show limited durability, with functional decline often occurring within two years. This creates an urgent need for more effective regenerative approaches in this active population. This study aimed to compare the short-term efficacy and safety of UC-MSCs injection alone and in combination with microfracture in this unique patient population.

## METHODS

A retrospective analysis was conducted on 32 patients with Outerbridge Grade-III or below articular cartilage injury of the knee with a defect area ≤ 2 cm^2^, who were admitted to the Department of Orthopedics in The General Hospital of North Theater Command Training Base from August 2020 to December 2020, and followed up for at least 12 months.

### Ethical approval:

The study was approved by the Institutional Ethics Committee of The General Hospital of North Theater Command Training Base (No.: Y(2020)010; Date: June 26, 2023), and written informed consent was obtained from all participants.

### Inclusion criteria:

- Diagnosis as articular cartilage injury of the knee, accompanied by pain and limited mobility.
- Aged 21-39 years old, and Outerbridge grade III and below articular cartilage injury of the knee with a defect area ≤ 2 cm^2^.
- Cartilage defects of the femoral condyle and trochlea caused by traumas or osteochondritis dissecans.

### Exclusion criteria:

- Weight exceeding 1.5 times the standard weight.^7^
- The inversion or eversion angle of the affected limb exceeding the normal value by 5° when standing.^8^,^9^
- Femoral stress axis located medial or lateral 1/4 outside the central tibial plateau.^8^
- Secondary osteoarthritis due to other diseases.
- Combined with other soft tissue injuries of the knee joint.
- Significant subchondral bone defects.

This clinical study was conducted in accordance with the ethical principles of the Declaration of Helsinki and global norms and regulations related to clinical drug trial management. The materials submitted prior to the study [research protocol, Case Report Form (CRF), Informed Consent Form (ICF), etc.] have been reviewed and approved by the Ethics Committee. Each participant and/or legal representative understands the purpose and procedures of the study and signs an informed consent form. UC-MSCs were prepared and provided by the Department of Experimental Medical Science in The General Hospital of North Theater Command Training Base. This retrospective cohort study included 32 military personnel with knee cartilage defects (≤2 cm², Outerbridge Grade-III or below),compared outcomes among three treatment groups, Group-A, B and C. Group-A (n=11) underwent microfracture alone; Group-B (n=10) received arthroscopic exploration + UC-MSCs injection (5×10^7^ cells in 5ml saline at one and four weeks post-arthroscopy); Group-C (n=11) received microfracture + UC-MSCs injection. Patients were matched across groups based on age (±3 years), defect size (±0.2 cm²), and Outerbridge grade. The baseline data of 32 patients is shown in Table-I. The surgical procedures of the three groups were all performed by the same senior physician with extensive clinical experience. The three groups showed no statistically significant differences in baseline data (P > 0.05), as seen in Table-I. All patients received outpatient follow-up according to a standardized follow-up protocol at three, six, nine and 12 months after treatment, and were evaluated using the Lysholm score and American Knee Society Score (AKSS). The evaluation method was the same for all groups.

**Table-I:** Comparison of preoperative general data among three groups of articular cartilage injury of the knee Concomitant injury.

Group	Sample size	Gender (male/female, n)	Age (year)	Affected side (left/right, n)	Body mass index	Meniscus	Anterior cruciate ligament	No
A	11	8/3	30.00±3.3	6/5	20.26±1.2	6	1	4
B	10	8/2	30.50±3.0	3/7	20.03±1.2	4	0	6
C	11	9/2	29.00±3.3	4/7	20.25±1.2	4	1	6
Fisher/F	-	0.443	0.593	1.423	0.121		2.297	
P	-	1.000	0.559	0.552	0.886		0.782	

UC-MSCs were isolated from human umbilical cord Wharton’s jelly using enzymatic digestion and expanded in vitro for 3-4 passages. Cell viability (>95%) and mesenchymal markers (CD73+, CD90+, CD105+, CD34-, CD45-, HLA-DR-) were confirmed before clinical use. Each UC-MSCs injection contained 5×10^7^ cells in 5ml normal saline, administered under aseptic conditions.

Group-A consisted of patients who undergo arthroscopic exploration and cleaning of the knee, and rest for two months after surgery to avoid strenuous exercise; Group-B consisted of patients who underwent arthroscopic exploration and clearance of the knee and received injection of UC-MSCs into the joint cavity at one and four weeks after one month. Group-C consisted of patients who underwent arthroscopic exploration, clearance, and microfracture of the knee and received injection of UC-MSCs into the joint cavity at one and four weeks after one month. Lysholm score and American Knee Society Score (AKSS) scores were collected for each case at three, six, nine, and 12 months of follow-up after treatment. microfracture and UC-MSC injection are shown in Fig.A, B and C.

**Fig.A:**
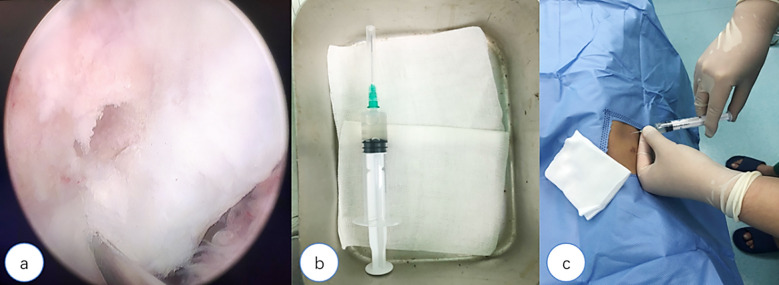
microfracture for lateral femoral condylar cartilage injury of the knee; Fig.B: Placement of UC-MSCs in a sterile iron box; Fig.C: UC-MSC injection into the knee joint cavity.

### Clinical outcome:

Demographics, injury characteristics (location/size via MRI and arthroscopy), Outerbridge grad; Surgical details documented in real-time by independent observers; Lysholm/AKSS assessments performed by blinded orthopedic specialists at dedicated clinics, and concomitant injuries (meniscus/ACL) were recorded. Outerbridge grading is used for knee cartilage injuries, therefore all included cases require arthroscopic examination. The AKSS scoring system is divided into two parts: knee scoring and functional scoring. It not only comprehensively evaluates the overall function and morphology of the knee joint, but also evaluates the joint’s own condition, effectively solving the problem of score decline caused by age-related diseases. The Lysol score focuses more on motor function, and the higher the score, the better the recovery of knee joint function.

### Statistical analysis:

Statistical analysis was carried out using SPSS 21.0.Continuous variables were tested for normality using Shapiro-Wilk test, normal distribution were expressed as mean ± standard deviation((*χ̅*±*S*). Comparisons used one-way ANOVA, and Kruskal-Wallis test with Dunn’s correction for non-normal data. Within-group changes were analyzed using paired t-tests or Wilcoxon signed-rank test as appropriate. The enumeration data were expressed as frequency and rate, and compared using the c^2^ test or Fisher’s exact probability test. P< 0.05 was considered statistically significant.

## RESULTS

In this study, two cases in groups B and C each experienced local fever and swelling in the knee joint after receiving stem cell injection therapy, while the remaining cases showed no significant abnormalities. All groups showed significant improvement in Lysholm scores from baseline to 12 months follow-up (all P<0.05, Table-II). Groups-B and C demonstrated superior outcomes compared to Group-A at six, nine, and 12 months (P<0.05), with no significant difference between them (P>0.05); there was no significant difference in Lysholm scores between the three groups before treatment. However, there were statistical differences in Lysholm scores during the follow-up at six, nine, and 12 months (P<0.05), The Lysholm scores of groups B and C were higher than those of Group-A, indicating better therapeutic effects, however, there was no statistically significant difference in Lysholm scores between Groups-B and C (t=0.735, 0.505, 0.387, P>0.05), indicating similar therapeutic effects. Table-II.

**Table-II:** Comparison of Lysholm score among the three groups at fixed time points during follow-up.

Group	n	Lysholm				
		Before treatment	After treatment (month)			
			3	6	9	12
A	11	51.00±1.6	63.00±3.3	68.64±2.1	74.00±3.3	74.64±2.4
B	10	51.50±2.2	62.00±2.6	71.80±1.9	79.50±3.6	79.40±4.3
C	11	50.91±2.4	60.55±2.6	71.09±2.4	78.64±4.3	78.73±4.2
F	-	0.242	2.051	6.233	6.791	5.800
p	-	>0.05	>0.05	<0.05	<0.05	<0.05

The AKSS functional scores of the three groups also showed significant improvement at the last follow-up compared to before treatment (p<0.05). There was no significant difference in AKSS knee score and AKSS functional score among the three groups before treatment. However, there were statistical differences in AKSS knee score and AKSS functional score during follow-up at six, nine, and 12 months after treatment (P<0.05), The AKSS knee score and AKSS functional score in Groups-B and C were higher than those in Group-A, and the difference was statistically significant (P<0.05), indicating better therapeutic effect. The therapeutic effects of Groups-B and C were similar (P>0.05,Table-III).

**Table-III:** Comparison of complications and AKSS among the three groups at fixed time points during follow-up.

	A	B	C	F	P
Complication Local swelling and fever	0	2	2	-	-
Lower-limb venous thrombosis	0	0	0	-	-
Infection	0	0	0	-	-
Joint stiffness AKSS knee score	0	0	0	-	-
Before treatment	47.27±7.5	46.50±7.5	47.73±7.3	0.071	>0.05
3 months	55.45±7.6	56.00±7.4	55.00±7.1	0.049	>0.05
6 months	67.27±5.2	73.00±5.9	72.27±5.2	3.582	<0.05
9 months	75.45±3.5	80.50±5.0	80.00±5.9	3.472	<0.05
12 months	75.00±3.9	80.50±5.0	81.36±5.0	6.005	<0.05
*AKSS functional score*					
Before treatment	50.00±3.5	50.60±3.5	50.55±3.8	0.093	>0.05
3 months	62.82±3.8	62.10±4.0	62.73±3.5	0.111	>0.05
6 months	68.64±2.1	72.80±3.4	72.82±3.1	7.240	<0.05
9 months	74.64±3.9	81.00±3.3	81.09±3.2	11.647	<0.05
12 months	75.18±3.3	81.60±4.2	80.18±3.8	8.478	<0.05

## DISCUSSION

In this study, UC-MSC injection was used to treat knee cartilage injury, and after nearly 12 months of follow-up, satisfactory therapeutic effects were achieved. Among the selected three groups, there were no significant inter-group differences in the Lysholm scores and AKSS of the knee before treatment. During the follow-up at six, nine and 12 months after treatment, the Lysholm scores and AKSS of the Groups B and C were higher than those of the Group-A, indicating higher efficacy. To the surprise of our research team, no significant differences were found in Lysholm scores or AKSS between the Group-B and the Group-C at different time points during follow-up, suggesting similar efficacy. Arthroscopic soft tissue repair or Microfracture can result in a certain degree of knee joint effusion, to reduce interference with the experimental results, Group-B and Group-C underwent arthroscopic surgery, and UC-MSC injection began one month later. In order to maintain consistency in time, Group-A patients need to rest for two months after surgery. During the three-month follow-up, there was no statistically significant difference in knee joint scores between the two groups. This may be due to the fact that umbilical cord mesenchymal stem cells (UC-MSCs) differentiation, maturation, and the crawling of fibrocartilage in the defect pool during Microfracture surgery require some time. The knee is one of the most important weight-bearing joints in the human body, military training often leads to knee joint injuries. Without prompt and accurate treatment, which seriously affect the recovery of the injured and cause adverse effects on the combat effectiveness of the military. Progressive articular cartilage degeneration is the central characteristic of osteoarthritis.^9^ Due to its insufficient blood and nutrient supply as well as its own cellular characteristics, transparent cartilage is almost unable to regenerate, and fibrocartilage is currently the main tissue source for repair.^10^

Currently, for patients with obvious symptoms of small-scale cartilage defects (injury area ≤ 2cm²), Microfracture or chondroplasty can be performed, which is simple to operate, low-cost, and has been used as the mainstream technique. But studies have shown that functional deterioration occurs two years after surgery, leading to an increased failure rate.^11^ Osteochondral Autograft Transfer System (OATS) requires the acquisition of non-load bearing cartilage for transplantation at the defect site, and it is difficult to match the donor cartilage with the lesion contour to form a uniform surface.^4^ Therefore, this technique are relatively rare in clinical practice. A 10 years follow-up study showed that only 34% of patients continued to participate in sports activities after receiving OATS treatment.^12^ For cartilage defects with an area > 2cm², there are currently many clinical treatments available, but no evidence to prove which treatment method is comprehensively superior. Osteochondral Allograft Transplants Surgery (OCA) is limited by low availability, loose connection between graft and wound margin, unevenness, a long waiting time, and a high cost.^13^ Matrix-induced Autologous Chondrocyte Implantation (MACI) was approved by the United States FDA in 2017. MACI presents promising postoperative outcomes compared with microfracture,^14^ This technology involves issues such as cell culture and stent placement, and has a long treatment cycle with uncertain efficacy, making it not widely used in clinical practice.

In recent years, stem cell-based cartilage repair has become a research hotspot in cartilage repair both domestically and internationally. as an important member of the stem cell family, MSCs have received increasingly widespread attention in that they not only have the potential for multi-directional differentiation and are involved in tissue regeneration and repair. Clinical research results have shown that stem cell therapy for KOA is safe, with no significant toxic side effects. Patients’ joint function and symptoms have been improved to a certain extent, clinical symptoms have been improved, and pain has been reduced or disappeared. The overall efficacy is satisfactory.^15^

Currently, adipose derived mesenchymal stem cells(AD-MSCs) and UC-MSCs are widely used in clinical practice,^16^ Koh et al.^17^ reported the application of AD-MSCs in treating articular cartilage defects of the knee, secondary arthroscopy revealed that only 24% of the patients had cartilage healing and repair to normal or near-normal levels, compared with other MSCs, AD-MSCs have lower chondrogenic potential.^18^ Due to the convenience of umbilical cord collection, no harm or damage to mothers and newborns, and few ethical controversies, UC MSCs are easy to isolate, have high purity, extremely low immunogenicity, great differentiation potential, and strong in vitro proliferation ability. In recent years, their application has become increasingly widespread. Steere et al.^19^ reported the use of UC MSCs for the treatment of KOA, and its effect is satisfactory.

Previous researchers have mostly used microfracture surgery for patients with obvious symptoms of small-scale cartilage defects, which utilizes fibrocartilage regeneration to alleviate pain to a certain extent, but the treatment effect is short-lived. Currently, there are few reports on the study of UC MSCs combined with microfracture surgery.^20^,^21^ Therefore, the research team applied microfracture surgery combined with UC MSCs knee joint injection to explore its clinical efficacy, and compared it with microfracture surgery and knee joint UC MSCs injection, aiming to prove that the combined approach is more excellent. After a one-year follow-up, B. Both groups C showed significant improvement compared to before treatment, and were superior to Group-A, which indirectly proves the role of UC MSCs. The standardized protocols for UC-MSCs preparation, surgical procedures, and outcome assessments ensure good reproducibility of our methods. The consistent cell viability (>95%) and phenotype characterization across batches support the reliability of our stem cell preparation process.

However, surprisingly, the combination of microfracture surgery and UC MSCs injection had similar therapeutic effects as simple UC MSCs knee joint injection. We believe that the reason for this phenomenon is that the differentiation effect of UC MSCs temporarily masks the role of microfracture surgery in the short term, so long-term follow-up is necessary. Finally, the gold standard for determining therapeutic efficacy is arthroscopic exploration and biopsy again, but it is extremely difficult to implement and therefore requires further exploration.

This study provides the first comparative evidence demonstrating that umbilical cord-derived mesenchymal stem cells (UC-MSCs) injection alone achieves comparable short-term clinical outcomes to combined microfracture with UC-MSCs therapy for small cartilage defects (≤2 cm²) in military personnel, challenging the traditional paradigm of routine microfracture and suggesting a potential shift toward less invasive regenerative strategies for active-duty soldiers. The study’s strengths include a standardized military cohort with carefully matched injury characteristics, blinded outcome assessments conducted by experienced orthopedic specialists, utilization of dual validated scoring systems (Lysholm and AKSS), and implementation of a reproducible UC-MSCs preparation protocol demonstrating consistent cell viability (>95%) and characteristic CD marker expression. Looking forward, future research should focus on long-term follow-up to assess treatment durability beyond two years, incorporate second-look arthroscopy with histological analysis for structural evaluation, perform cost-effectiveness analyses comparing UC-MSCs versus microfracture approaches, and conduct multicenter trials with larger sample sizes to further validate these findings.

### Limitations:

Firstly, the number of collected samples is relatively small. second, there is a lack of secondary arthroscopic evaluation on the experimental subjects after injecting stem cells. Thirdly, the follow-up time is short fourth, stem cell therapy is expensive. Furthermore, its immune rejection and other side effects are not fully understood.

## CONCLUSION

In summary, simple microfracture surgery can achieve certain therapeutic effects for small-scale cartilage defects. Compared with expensive stem cell injection, microfracture surgery still has its advantages. In short-term follow-up, simple UC MSCs injection or microfracture surgery combined with UC MSCs injection can achieve more satisfactory therapeutic effects compared to microfracture surgery. From the perspective of knee score or functional score, simple UC MSCs injection can achieve the same therapeutic effect as microfracture surgery combined with UC MSCs injection, so microfracture surgery is not necessary.

### Authors’ Contributions:

**GZ** and **CX:** Carried out the studies, participated in collecting data, and drafted the manuscript, and are responsible and accountable for the accuracy or integrity of the work.

**BL** and **WJ:** Performed the statistical analysis and participated in its design. Literature seach.

**XL:** Acquisition, analysis, interpretation of data and draft the manuscript.

All authors have read and approved the final manuscript.
